# Evaluation of Rearing Factors Affecting *Clanis bilineata tsingtauica* Mell Larvae Fed by Susceptible Soybean Variety NN89-29 in Spring and Autumn Sowing

**DOI:** 10.3390/insects14010032

**Published:** 2022-12-29

**Authors:** Xiaofeng Liu, Yulu Yan, Nan Liu, Yufei Xu, Huiyan Jiang, Zhihao Ye, Hao Wang, Junyi Gai, Guangnan Xing

**Affiliations:** Soybean Research Institute, MARA National Center for Soybean Improvement, MARA Key Laboratory of Biology and Genetic Improvement of Soybean, National Key Laboratory for Crop Genetics and Germplasm Enhancement, Jiangsu Collaborative Innovation Center for Modern Crop Production, Nanjing Agricultural University, Nanjing 210095, China

**Keywords:** soybean, *Clanis bilineata tsingtauica* Mell, sowing season, rearing factor, edible insect

## Abstract

**Simple Summary:**

Edible insects have the advantages of shorter feeding cycles, lower cost and lower environmental burden than traditional meats. So, they have gradually become a complement to traditional animal protein in recent years. The larvae of *Clanis bilineata tsingtauica* Mell (Lepidoptera: Sphingidae) commonly known as Doudan in China, is a common leaf-eating pest on soybeans and a nutritious edible insect. This study evaluated the effects of different rearing factors on growth of *C. bilineata tsingtauica* larvae and clarified the factors and optimal levels of artificial rearing.

**Abstract:**

*Clanis bilineata tsingtauica* Mell is a nutritious edible insect. In the present study, soybean variety NN89-29 susceptible to leaf-feeding insects was used as the experiment material to evaluate the rearing factors affecting the production of *C. bilineata tsingtauica* in spring and autumn sowing. We artificially inoculated *C. bilineata tsingtauica* eggs to soybean plants, and the relevant indexes of larvae and soybean plants were recorded. The main results from spring sowing were as follows: (1) Larval number, single larval weight and plot-larval weight were all higher in the V6 stage (sixth trifoliolate) than those in the R3 stage (beginning pod) of inoculated eggs. (2) Larval number, single larval weight and plot-larval weight significantly decreased under higher planting density. Meanwhile, the soybean plant height and internode length increased, while the main stem node number of soybean decreased under higher planting density. (3) Single larval weight and plot-larval weight were not significantly affected by different numbers of eggs inoculated, but the larval number was significantly affected. Under autumn sowing conditions: (1) The larvae production and soybean plant growth were not significantly affected by covering the top of the net house with plastic film to isolate rainwater. (2) More eggs inoculated were associated with higher plot-larval weight. Conversely, fewer eggs inoculated resulted in a higher proportion of single larvae weight more than or equal to 6 g (≥6 g). Compared to spring sowing, the low biomass of autumn-sown soybean plants did not provide sufficient food for *C. bilineata tsingtauica* growth, and competition for food resulted in lower larval number, single larval weight and plot-larval weight.

## 1. Introduction

Rapidly growing populations have created an urgent need for humans to seek potential food sources to ensure food security. Insects have a short rearing cycle, high food conversion efficiency and high protein content [1,2], which can be used as an important supplement for human protein sources [3]. Along with the emphasis on the value of edible insects, the market for edible insects is expanding year after year. The global edible insect market value was predicted to increase to over USD 1.18 billion by 2023 [4]. Insects, containing high nutritional value, are rich in protein, fat and minerals. In general, the protein content of insects ranges from 40% to 75% dry weight (DW), the fat content from 7% to 77% DW and the mineral content from 3% to 8% DW [5,6,7]. Insects are also rich in various vitamins [8], becoming good vitamin supplements.

The active substances in insects are beneficial to animals and human health due to their potential health value [9]. The black soldier fly (*Hermetia illucens* Linnaeus) produces inducible antibacterial peptides that have in vitro activity against *Helicobacter pylori* (Marshall and Goodwin) [10]. Two homologous insects CSαβ peptides-DLP2 and DLP4 from *Hermetia illucens* display potent antimicrobial activity against Gram-positive bacteria especially methicillin-resistant *Staphylococcus aureus* [11]. Chitosan, extracted from insects such as the mealworm beetle, has a broad antimicrobial spectrum to Gram-negative and Gram-positive bacteria and fungi [12,13].

*Clanis bilineata tsingtauica* Mell, known as soybean hawkmoth in China, is reported to have one to two generations every year [14]. As a leaf-chewing insect, the larvae mainly feed on plants such as soybean, acacia and velvetbean [14]. The larval development includes five instars, and the food intake of the larval gluttonous stage (the fifth instar) can account for about 90% of the whole growth period [15].

*Clanis bilineata tsingtauica* has a long history as an edible insect in China and is currently consumed in several provinces of China, such as Jiangsu, Shandong and Henan. The meat of *C. bilineata tsingtauica* larvae is used freeze-dried, fried, fresh and canned [16]. The larva is a high-quality protein source, which has a high content of protein, essential amino acids and essential fatty acids [17]. Sixteen different amino acids were quantified in *C. bilineata tsingtauica* larvae, and the larva proportion of essential amino acids in total amino acids was 53%, which is higher than in eggs (51%), milk (47%) and soybeans (44%) [14]. The crude protein content of larvae was 66%, crude fat content was 24% and unsaturated fatty acids were 64%, of which linolenic acid was 37% [17]. Among unsaturated fatty acids, linolenic acid shows a high nutritional and medical value, with the effect of lowering blood lipids and reducing the incidence of cancer [18]. Fe in the epidermis and Zn in the meat were abundant at 164 and 299 µg/g of dry weight, respectively [17]. The algal polysaccharides contained in the larvae are about ten times higher than those in mammals [19]. The algal polysaccharides can stimulate macrophages, improve immune response, inhibit the proliferation of cancer cells and protect neural cells [20,21]. In addition, *C. bilineata tsingtauica* can be used in aquaculture. Oral administration of the larvae in fishmeal diets improved shrimp growth performance, body composition and immunity status [22].

The rearing of *C. bilineata tsingtauica* started in Lianyungang city and the surrounding areas in Jiangsu province of China. The market and the range of rearing are expanding year by year and have spread to Shandong, Henan, Anhui and Hubei provinces. The larvae rearing is mainly carried out in greenhouses and net houses [23,24]. At present, professional farmers are generally able to raise two generations of *C. bilineata tsingtauica* per year. The first generation of larvae, fed by spring-sown soybeans in greenhouses, are generally marketed from June to July each year, while the second generation of larvae fed by summer-sown soybeans are generally marketed from August to September [25]. To improve the hatching rate and larval survival of *C. bilineata tsingtauica* eggs, placing plastic film and sunshade board on the net cage has proven effective [26]. Different soybean planting densities have a significant effect on the growth and development process as well as the final yield of the *C. bilineata tsingtauica* larvae [27]. Meanwhile, different numbers of eggs inoculated significantly affect larval production and soybean yield [28]. Li et al. planted soybeans in the same plot in mid-February, early May and late July by building greenhouses and spreading mulch, achieving three generations of *C. bilineata tsingtauica* in a year with an average production value of more than CNY 150,000 per hectare [29].

Xing et al. selected soybean variety NN89-29 which is susceptible to *Spodoptera litura* [30]. Therefore, we hypothesize that this soybean variety is also susceptible to *C. bilineata tsingtauica* larvae and is suitable for feeding the larvae. Among reported studies, there is a lack of comprehensive research on the factors affecting larvae development in spring and autumn sowing, which limits the expertise available. To improve the yield, the present study used insect susceptible soybean variety NN89-29 to feed the larvae and evaluated the relevant factors affecting rearing by using net houses in the spring and autumn sowing seasons with the aim of: (1) exploring the effects on larval production of the soybean growth stage when eggs were introduced, the number of eggs inoculated, planting density, covering plastic film and the interactions between these factors; (2) comparing the differences of larval rearing between spring and autumn; and (3) synthesizing the appropriate levels of various factors influencing larval rearing and proposing technical conditions for high-yield larvae rearing in spring and autumn sowing, respectively.

## 2. Materials and Methods

### 2.1. Test Insects

The eggs of *C. bilineata tsingtauica* were obtained from the professional rearing base in Lianyungang, Jiangsu Province, China. Eggs laid on the same day were surface sterilized and used in experiments.

### 2.2. Experimental Conditions and Materials

Experiments were performed at the Wanjiang Base (118°37′22″ E and 31°33′4″ N) of Nanjing Agricultural University, Dangtu County, Ma’anshan City, Anhui Province, China. Temperature, humidity and light intensity were recorded by sensors installed in a soybean field, and the duration of sunshine was recorded by the local meteorological station. Herbicide was sprayed once in the net houses before soybean sowing to prevent weeds from adversely affecting the test. Insecticides were prohibited during the experiments. In the experiments, the net houses (2 m × 2 m) were all spliced with a steel pipe skeleton structure covered with white nylon mesh.

### 2.3. Evaluation of C. bilineata tsingtauica Rearing Factors under Spring Sowing Condition

**Figure 1:**
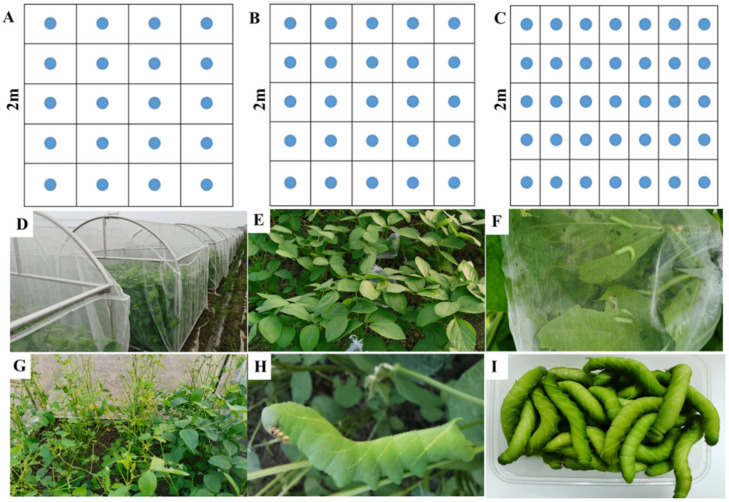
The cultivation mode of the soybean and *C. bilineata tsingtauica* rearing process in net houses. (**A**–**C**): soybean sowed in a 2 m × 2 m net house in spring sowing, blue circles represent soybean plants (hills); (**A**) 0.4 m × 0.5 m row spacing; (**B**) 0.4 m × 0.4 m row spacing; (**C**) 0.4 m × 0.3 m row spacing; (**D**) soybean was grown in net houses; (**E**) soybean leaves were bagged to inoculate eggs; (**F**) larvae fed on leaves in the mesh bag (15 cm × 20 cm); (**G**) soybean plants were fed on freely by larvae; (**H**) larvae habitually clung to the soybean petiole; (**I**) larvae harvested.

NN89-29, a soybean variety susceptible to leaf-feeding insects, was used to evaluate *C. bilineata tsingtauica* rearing factors. The experiment was arranged in a randomized block design, and treatments consisted of a 2 × 2 × 3 factorial. The twelve treatments were composed of combinations of two soybean growth stages (V6 stage, sixth trifoliolate; R3 stage, beginning pod) [31], two quantities of eggs inoculated (75 and 100 eggs per net house, i.e., 18.75 eggs/m^2^ and 25 eggs/m^2^) and three planting densities (approximately 15 plants/m^2^, 19 plants/m^2^ and 26 plants/m^2^). Two replications were used per treatment. Soybeans were sown in hills on 28 April 2021, each plot corresponding to a 2 m × 2 m net house. Under planting densities of 15 plants/m^2^, 19 plants/m^2^ and 26 plants/m^2^, the row spacing × column spacing of soybean plants was 0.4 m × 0.5 m, 0.4 m × 0.4 m and 0.4 m × 0.3 m, respectively (Figure 1A–C). Then, the seedlings were thinned to three plants in each hill plot to make the planting density in each net house accurate.

In this experiment, two quantities of eggs inoculated (75 and 100 eggs per net house) were adopted. Mesh bags (15 cm × 20 cm) with one side opening and drawstring were used to pick up the eggs, 15 and 20 eggs per mesh bag corresponding to 75 and 100 eggs per net house (i.e., 18.75 eggs/m^2^ and 25 eggs/m^2^), respectively. We stuffed the third trifoliolate from the soybean plant apex into the bag containing the eggs to inoculate the eggs. Every mesh bag was attached in the middle of each row, and five bags of eggs were hung in each net house. The artificial inoculation with *C. bilineata tsingtauica* eggs for the V6 stage soybean plants was carried out on 4 June. These eggs hatched on 8 June. Then, the 5-day-old larvae were released from the mesh bag on 12 June and were allowed to feed freely in the net houses (Figure 1E–G). The larvae were harvested on 1 July (Figure 1I). The R3 stage plants were inoculated artificially with eggs on 30 June. The eggs hatched on 1 July, and the larvae were released to the net house on 5 July. The larvae were harvested on 24 July (Figure 1I). Larvae habitually clung to the soybean petiole after they were grown, making them relatively easy to detect and catch (Figure 1H). All these larvae were removed from the net houses, counted and weighed. After every weighing, the main stem node number and plant height of soybean plants were recorded in each net house. A five-point sampling method was used, and five plants were measured in each net house.

### 2.4. Evaluation of C. bilineata tsingtauica Rearing Factors under Autumn Sowing Condition

The highly susceptible soybean variety NN89-29 was chosen to feed the larvae. The experiment was also carried out in 2 m × 2 m net houses, arranged in a randomized block design, and treatments consisted of a 2 × 3 factorial. The six treatments were conducted with the net houses either uncovered or covered with plastic film and three quantities of eggs inoculated (15 eggs/m^2^, 20 eggs/m^2^ and 25 eggs/m^2^). Three replications were used per treatment. Soybeans were sown on 5 August. Row spacing × column spacing was 0.4 m × 0.5 m (5 rows and 4 columns), and the seedlings were thinned to 3 plants in each hill. Twenty eggs were placed in each mesh bag (15 cm × 20 cm). Three, four and five bags of eggs were distributed in each net house corresponding to the three quantities of eggs inoculated, respectively. Mesh bags were hung separately on leaves to artificially inoculate on 13 September. Larvae were released on 24 September and were adjusted uniformly based on hatchability among replicates of the same treatment. All larvae within each net house were caught, counted and weighed individually on 10 October. The main stem node number and plant height of soybean plants was recorded in net house. A five-point sampling method was used, and five plants were measured in each net house.

### 2.5. Statistical Analyses

The growth of *C. bilineata tsingtauica* larvae and the agronomic traits of soybeans in the net houses in the spring sowing were analyzed by three-way analysis of variance (ANOVA). Additionally, a two-way analysis of variance (ANOVA) was used to analyze the larval growth of *C. bilineata tsingtauica* and the agronomic traits of soybeans in the net houses in the autumn sowing. The unpaired *t*-test was used to compare the meteorological factors between spring and autumn. Data were stored in Microsoft Excel (2013), and analysis of variance, multiple comparisons and correlation analysis were analyzed with SAS 9.4 statistical software. PROC GLM process was used for ANOVA, and Duncan’s method was used for multiple comparisons. The internode length was calculated by the formula: internode length = plant height/main stem node number.

## 3. Results

### 3.1. Evaluation of C. bilineata tsingtauica Rearing Factors under Spring Sowing Condition

#### 3.1.1. Effects of Rearing Factors on the Growth of *C. bilineata tsingtauica* and Soybean Plants in Spring Sowing

Larval number was significantly affected by the growth stages of the soybean plants to inoculate eggs (*p* < 0.05). Single larval weight and plot-larval weight were highly significantly affected by the growth stages of the soybean plants to inoculate eggs (*p* < 0.01). Larval number, single larval weight and plot-larval weight were extremely significantly influenced by the soybean planting density (*p* < 0.01). The number of eggs inoculated had a significant effect only on larval number. The interaction of growth stage and planting density significantly affected single larval weight and had an extremely significant effect on plot-larval weight (Table 1). Other interactions had no significant effect on larval number, single larval weight and plot-larval weight (Table 1).

Soybean growth stages to artificially inoculate eggs significantly influenced plant height, main stem node number and internode length (*p* < 0.01) (Table 2). Furthermore, significant differences were observed in plant height, main stem node number and internode length of the soybean plants among different planting densities (*p* < 0.01). There was no significant difference in agronomic traits of the soybean plants between different quantities of eggs inoculated. Agronomic traits of the soybean plants were not significantly affected by the interactions between/among rearing factors (Table 2).

**Figure 2:**
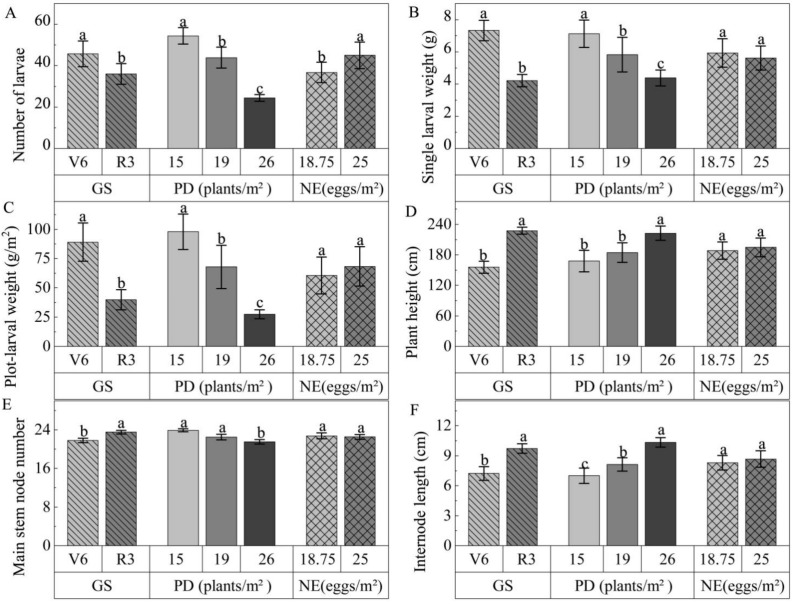
Differences of larval growth indicators and soybean agronomic traits between/among different levels of various factors in spring sowing net houses: (**A**) number of larvae; (**B**) single larval weight; (**C**) plot-larval weight; (**D**) plant height; (**E**) main stem node number; (**F**) internode length; GS, growth stage; PD, planting density; NE, number of eggs inoculated; different lowercase letters between/among levels of rearing factors indicate significant differences of *p* < 0.05.

Larval number, plot-larval weight and single larval weight were significantly higher in the soybean V6 stage to inoculate eggs than those in the R3 stage (*p* < 0.05), while soybean plant height, main stem node number and internode length in the V6 stage to inoculate eggs were significantly lower than those in the R3 stage (Figure 2). The soybean plants at 15 plants/m^2^ had the most larvae, plot-larval weight and single larval weight. Those of the soybean plants at 19 plants/m^2^ were next, and those of the soybean plants at 26 plants/m^2^ were the lowest. That is, as plant density increased, these larval growth indicators decreased, and the three *C. bilineata tsingtauica* larval growth indicators were significantly different among the three planting densities (Figure 2A–C). At the same time, soybean plant height was significantly lower, and the main stem node number was significantly higher at 15 and 19 plants/m^2^ than at 26 plants/m^2^ planting density. The soybean internode length was shortest (7.0 cm) at 15 plants/m^2^, next longer at 19 plants/m^2^ and longest (10.3 cm) at 26 plants/m^2^, with significant differences among these three planting density levels (Figure 2D–F) (*p* < 0.05). The larval number in the net houses with 25 eggs/m^2^ (100 eggs per net house) was significantly higher than that in the net houses with 18.75 eggs/m^2^ (75 eggs per net house) (*p* < 0.05), but there was no significant difference in single larval weight, plot-larval weight, plant height, main stem node number and internode length (Figure 2).

#### 3.1.2. The Relationship of Growth Indicators of *C. bilineata tsingtauica* and Soybean Plant

The correlation analysis indicated that larval number in the net houses was significantly positively correlated with single larval weight (*r* = 0.83; *p* < 0.05) and with plot-larval weight (*r* = 0.98; *p* < 0.01), while larval number in the net houses was highly significantly negatively correlated with plant height (*r* = −0.94; *p* < 0.01) and internode length (*r* = −0.94; *p* < 0.01) in the V6 stage. Single larval weight was extremely significantly positively correlated with plot-larval weight (*r* = 0.93; *p* < 0.01) and was significantly positively correlated with main stem node number (*r* = 0.82; *p* < 0.05), while single larval weight was extremely significantly negatively correlated with plant height (*r* = −0.93; *p* < 0.01) and internode length (*r* = −0.94; *p* < 0.01) in the V6 stage. Plot-larval weight was significantly negatively correlated with plant height (*r* = −0.98; *p* < 0.01) and internode length (*r* = −0.99; *p* < 0.01) and was significantly positively correlated with main stem node number (*r* = 0.86; *p* < 0.05) in the V6 stage. Plant height was significantly negatively correlated with the main stem node number (*r* = −0.85; *p* < 0.05) but was extremely significantly positively correlated with internode length (*r* = 0.99; *p* < 0.01) in V6 stage. Main stem node number was significantly negatively correlated with internode length (*r* = −0.89; *p* < 0.05) in the V6 stage (Table 3). As in the V6 stage, the growth traits of larvae were positively correlated in the R3 stage; the growth traits of larvae were also negatively correlated with soybean plant height and internode length in the R3 stage; soybean plant height (*r* = −0.96; *p* < 0.01) and internode length (*r* = −0.98; *p* < 0.01) were also negatively correlated with the main stem node number in the R3 stage.

#### 3.1.3. Comprehensive Effects of *C. bilineata tsingtauica* Rearing Treatments in Spring Sowing

The treatment at 15 plants/m^2^ with 25 eggs/m^2^ in the V6 stage had the highest larval number and plot-larval weight, with the plot-larval weight and single larval weight reaching 130.8 g/m^2^ and 8.1 g, respectively. The highest average single larval weight of the treatment at 15 plants/m^2^ with 18.75 eggs/m^2^ in the V6 stage was 9.4 g, and the plot-larval weight was up to 124.8 g/m^2^. Additionally, these two treatments had the lowest plant height and the shortest internode length, and they were the best two treatment combinations tested. The number of larvae and the plot-larval weight were the smallest in the treatment with 18.75 eggs/m^2^ at 26 plants/m^2^ in the R3 stage, with the plot-larval weight only reaching 17.1 g/m^2^. The lowest single larval weight was only 3.3 g in the treatment with 25 eggs/m^2^ at 26 plants/m^2^ in the R3 stage, and plant height and internode length were the highest (Table 4). This treatment was the most unsuitable for *C. bilineata tsingtauica* rearing.

### 3.2. Evaluation of C. bilineata tsingtauica rearing factor in Autumn Sowing

Extremely significant differences were found in larval numbers among different egg inoculation levels (*p* < 0.01). Single larval weight and plot-larval weight were significantly different among egg inoculation levels (*p* < 0.05) (Table 5). Covering the net houses with plastic film had no significant effect on the larval number, single larval weight and plot-larval weight (Table 5). The different numbers of egg inoculation and plastic film on top of the net house had no significant effect on plant height, main stem node number and internode length of soybean plants (Table 6).

**Figure 3:**
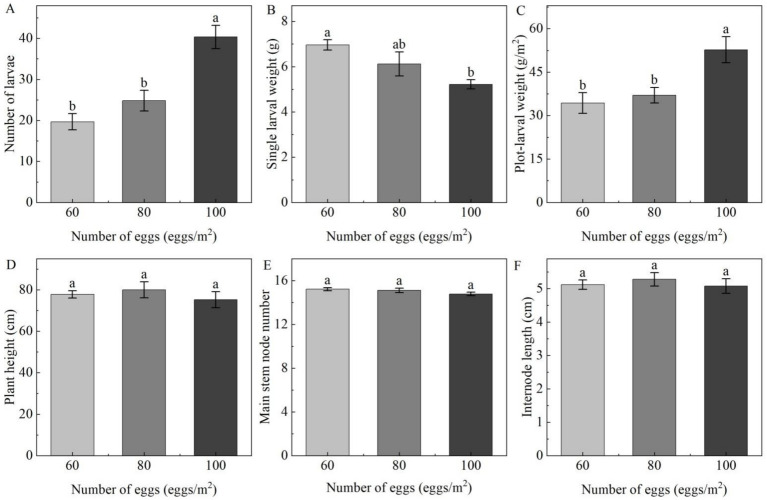
Multiple comparisons of growth indicators of *C. bilineata tsingtauica* larvae and soybean plants among different quantities of eggs inoculated in autumn sowing: (**A**) number of larvae; (**B**) single larval weight; (**C**) plot-larval weight; (**D**) plant height; (**E**) main stem node number; (**F**) internode length; different lowercase letters among different egg inoculation levels indicate significant differences of *p* < 0.05.

The lowest number of larvae was recorded under 15 eggs/m^2^, with maximum single larval weight of 7.0 g and the lowest plot-larval weight 34.3 g/m^2^, while the highest larval number and lowest single larval weight 5.3 g were recorded under 25 eggs/m^2^, with maximum plot-larval weight of 52.8 g/m^2^ (Figure 3). Larval number and plot-larval weight with 25 eggs/m^2^ inoculated density were significantly higher than those with 15 eggs/m^2^ and 20 eggs/m^2^ eggs inoculated density in autumn sowing (Figure 3A,C). In contrast, the single larval weight with 15 eggs/m^2^ inoculated density was significantly higher than that with 25 eggs/m^2^ inoculated. There was no significant difference between 20 eggs/m^2^ and 25 eggs/m^2^ (Figure 3B). In addition, plant height, internode length and main stem node number were not significantly influenced by the number of eggs inoculated in autumn sowing (Figure 3E,F).

**Figure 4:**
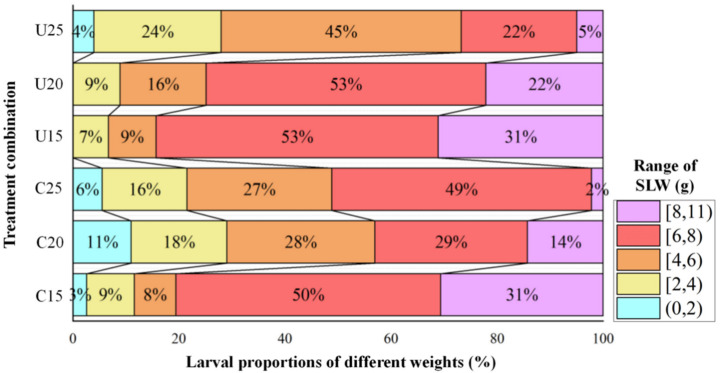
Interval distribution of single larval weight of different rearing treatments of NN89-29 in autumn sowing. U25, U20 and U15 are without plastic films on top of the net houses, with 25 eggs/m^2^, 20 eggs/m^2^ and 15 eggs/m^2^, respectively. C25, C20 and C15 are with plastic film covering the net houses, with 25 eggs/m^2^, 20 eggs/m^2^ and 15 eggs/m^2^, respectively. The legend numbers on the right-side refer to the range of single larval weight. The blue, yellow, orange, red and purple color blocks represent single larval weight of (0, 2) g, [2, 4) g, [4, 6) g, [6, 8) g and [8, 11) g, respectively.

The proportion of *C. bilineata tsingtauica* single larvae weighing more than or equal to 6 g (≥6 g) in each net house was significantly affected by the number of eggs inoculated, while covering plastic film on the top of the net house had no significant effect on the larval proportion of single larval weight more than or equal to 6 g (≥6 g) in each plot (Table 5). The lower the number of eggs inoculated, the greater the larval proportion weighing more than or equal to 8 g (≥8 g) in each plot was found. The highest larval proportion weighing more than or equal to 6 g (≥6 g) in the plot was found at 15 eggs/m^2^, with exceeding 80% (Figure 4).

### 3.3. Comparison of Rearing Results between Spring and Autumn Sowing

**Figure 5:**
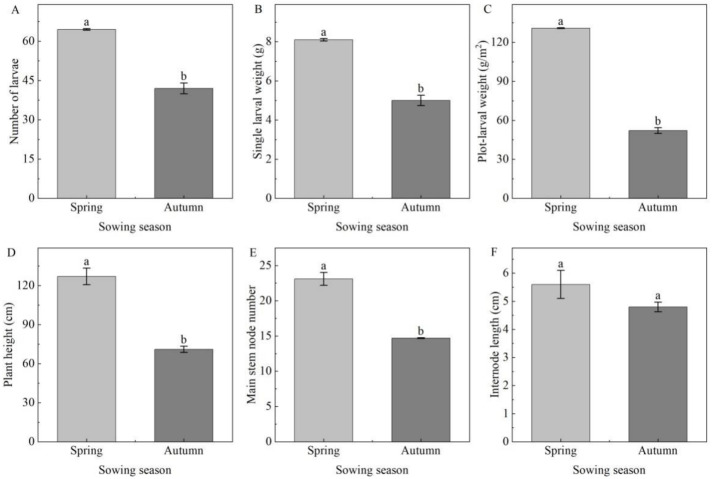
Comparison of growth indicators of *C. bilineata tsingtauica* larvae and soybean plants between spring and autumn sowing: (**A**) number of larvae; (**B**) single larval weight; (**C**) plot-larval weight; (**D**) plant height; (**E**) main stem node number; (**F**) internode length. Different lowercase letters between spring and autumn sowing indicate significant differences of *p* < 0.05.

The results of *C. bilineata tsingtauica* larvae rearing and soybean plant growth with the same planting density, plant growth stage to inoculate eggs and egg inoculation number in spring and autumn sowing showed that larval number, single larval weight, plot-larval weight, plant height and main stem node number of autumn sowing decreased significantly, and internode length also decreased somewhat (Figure 5).

**Figure 6:**
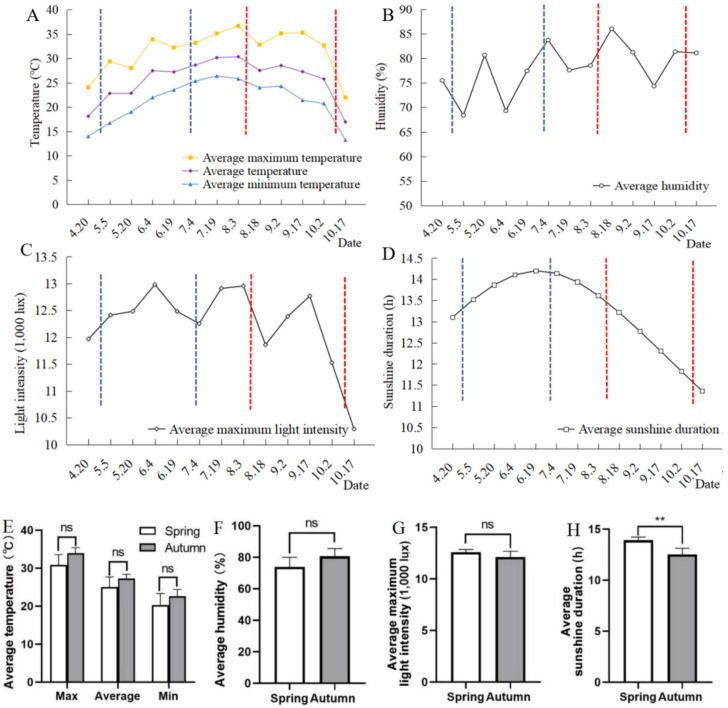
Average meteorological factor every 15 days of spring and autumn sowing. (**A**–**D**) Trend chart of average meteorological factors every 15 days for spring and autumn sowing: (**A**) temperature; (**B**) humidity; (**C**) light intensity; (**D**) sunshine duration; the insect rearing duration in the V6 stage to inoculate eggs in spring sowing is between the blue dotted lines, and the insect rearing duration in autumn sowing is between the red dotted lines. (**E**–**H**) Comparison of meteorological factors between spring sowing and autumn sowing: (**E**) temperature; (**F**) humidity; (**G**) light intensity; (**H**) sunshine duration; the meteorological factors of the two sowing seasons in spring and autumn were compared by *t*-test; ns: not significant; ** represents significance at 0.01 probability level.

The averages of maximum temperature, average temperature and minimum temperature per fifteen-day period increased gradually in spring, while they decreased gradually in autumn (Figure 6A). However, no significant difference was observed between the spring and autumn season (Figure 6E). The average humidity and average maximum light intensity of the spring sown and autumn sowing were similar (Figure 6B,C), and no significant difference was found between the two seasons (Figure 6F,G). The average sunshine duration in autumn was 12.5 h, which was significantly lower than that (13.9 h) in spring (Figure 6D,H). Temperature, humidity, light intensity and sunshine duration affected the growth of soybeans and *C. bilineata tsingtauica* larvae, which needs further research.

## 4. Discussion

### 4.1. Effects of Different Soybean Growth Stages to Inoculate Eggs on the Production of C. bilineata tsingtauica in Spring Sowing

In the present experiment, single larval weight and plot-larval weight in the soybean R3 stage were lower than those in the soybean V6 stage in spring sowing. The result could be explained by the excessive growth of the plants when inoculated with eggs in the R3 stage. When the larvae were harvested, the plant height, main stem node number and internode length of soybean plants at 15 plants/m^2^ in the R3 stage to inoculate eggs reached or even exceeded those at 26 plants/m^2^ in the V6 stage to inoculate eggs. Larvae feeding on soybean plants inhibited excessive plant growth, reduced plant lodging and increased ventilation and light transmittance in the net houses. In this way, the soybean plants artificially inoculated with eggs in the V6 stage were more suitable for larval growth.

Several reports have shown that insects grow differently after feeding on different growth stages of plants. The development of the velvetbean caterpillar, *Anticarsia gemmatalis* Hübner (Lepidoptera: Erebidae) and the fall armyworm, *Spodoptera frugiperda* (J.E. Smith) (Lepidoptera: Noctuidae), was negatively affected when larvae fed on leaves of the resistant genotype, older leaves from the lower part of plants or leaves from reproductive-stage soybeans [32]. Distribution and concentrations of nutrients and flavonoids of leaf age and plant stage in soybeans may explain the varying levels of antibiosis to the velvetbean caterpillar and the fall armyworm [32].

### 4.2. Effects of Different Soybean Planting Densities on the Production of C. bilineata tsingtauica in Spring Sowing

Compared with low planting density (16.7 plants/m^2^), high soybean planting density (33.3 plants/m^2^) resulted in longer larval development and less larval number in summer sowing, with lower quality and yield of *C. bilineata tsingtauica* larvae [27]. Excessive planting density will aggravate the lodging and winding of plants and affect ventilation and light transmission conditions in the field, with thinner stems, higher plant height and longer internodes and fewer nodes [33].

The present experiment found that the increase of soybean planting density led to a significant increase in plant height and internode length, with a decrease of main stem node number and larval yield. Overgrown soybean plants, with a height of more than 1.5 m, are more likely to have excessive elongation and lodging and to interfere with ventilation and light transmission in net houses. This will cause premature senescence of the leaves at the lower part of plant, which is not conducive to the growth of *C. bilineata tsingtauica* larvae.

### 4.3. Effects of Different Numbers of Eggs Inoculation on the Production of C. bilineata tsingtauica in Spring and Autumn Sowing

Single larval weight and plot soybean yield decreased and the number of empty soybean pods increased with increasing numbers of *C. bilineata tsingtauica* larvae inoculation [28]. The present result also indicated that the reasonable combination of the number of eggs inoculated and the biomass of soybean plants was an important factor to achieve high-yielding *C. bilineata tsingtauica* larvae. The difference of larval yield between the numbers of eggs inoculated in spring sowing was insignificant in the present study. Different egg inoculations significantly affected larval rearing in autumn sowing. Interestingly, the same trends were observed in spring sowing and autumn sowing: At the same planting density, more eggs inoculated resulted in more larvae and plot-larval weight, but increased competition for food led to lower single larval weight.

### 4.4. Effects of Covering Film on the Top of Net Houses on the Production of C. bilineata tsingtauica Rearing

The hatching rate of eggs and the survival rate of larvae were improved by rearing *C. bilineata tsingtauica* larvae in a cage that can protect them from rain and strong light in the field [26]. In this experiment, plastic film covering the top of the net house had no significant effect on the production of larvae in autumn sowing. It may be that the rain was less in the autumn, which led to the insignificant rain-blocking function of the film covering. In addition, only the tops of the net houses were covered, which hardly changed the temperature and humidity conditions inside the net house. Thus, further research is needed on the effect of plastic film coverings on *C. bilineata tsingtauica* rearing in net houses.

### 4.5. Effects of Sowing Season on the Production of C. bilineata tsingtauica

Compared with the test in spring sowing, single larval weight, soybean plant height and main stem node number were relatively small in autumn sowing. For instance, the main stem node number was more in spring sowing than in autumn sowing. So, it seems possible that these results were due to insufficient plant growth in autumn sowing, which could not provide sufficient food for *C. bilineata tsingtauica* larvae. Soybeans sown in autumn flowered in advance and only had short vegetative growth. In contrast, those sown in spring tended to delay flowering and had long vegetative growth. In addition, the temperature under autumn sowing gradually decreased, which might also be detrimental to the growth of larvae. Therefore, sowing in autumn should be started appropriately in advance, and planting density should be increased to obtain higher yield of *C. bilineata tsingtauica* larvae.

### 4.6. Other Rearing Factors Affecting the Production of C. bilineata tsingtauica

Temperature and humidity also have an extremely important impact on the growth and development of larvae. The duration of diapause and the pupal stage of *C. bilineata tsingtauica* gradually shortened as temperature increased; the adult emergence rate decreased significantly with increasing temperature [34]. The egg hatching rate of *C. bilineata tsingtauica* is greatly affected by temperature and humidity, and 30°C and 50% relative humidity are the most suitable conditions to hatch eggs [35]. The effect of temperature and humidity on the growth of *C. bilineata tsingtauica* is still unclear and needs further study. Meteorological factors such as temperature and humidity are related to the rearing location. We evaluated the rearing effect of using NN89-29 in Dangtu County, Anhui Province, 394 km southwest of Lianyungang in spring and autumn in the present study. At similar rearing times, when other soybean varieties were used to feed the *C. bilineata tsingtauica* larvae, the maximum plot-larval weight was about 94.5 g/m^2^ [27,28], which was far less than the maximum plot-larval weight in the present study (130.8 g/m^2^). This shows that the NN89-29 soybean variety is indeed susceptible to the *C. bilineata tsingtauica* larvae. Rearing evaluations in the area around Lianyungang will meet the expanded market demand for the larvae. The storage time of *C. bilineata tsingtauica* eggs can be extended to provide a stable supply of eggs through storage at 15 °C for 11 days and then held at 15–20 °C under dark conditions [36].

### 4.7. Challenges and Prospects

Although the present study obtained a relatively high yield of *C. bilineata tsingtauica* larvae in spring sowing, the main rearing factors and their levels maybe different under different conditions. The *C. bilineata tsingtauica* larval development duration when raised on the late-maturing varieties was longer than that under the mid-early maturing varieties at the same sowing time, and the yield and survival rate of the larvae raised mid-maturing soybean varieties were better than those of early-maturing and late-maturing varieties [27]. So, the optimal number of eggs inoculated and plant density should be selected by combining the local climatic conditions, sowing season and soybean variety.

Expanding the market demand is also necessary to generate more economic and social value for increasing the yield of *C. bilineata tsingtauica* larvae. Clever marketing and taste proved effective to convince people to eat insects [37]. What is more, *C. bilineata tsingtauica* larvae is generally considered to be safe with a long history of consumption in China, while toxicological assessment, allergens and pollutants should be considered for the evaluation process [38]. All the above points should be taken into account for the sustainable development of the *C. bilineata tsingtauica* industry.

## 5. Conclusions

Whether it is spring or autumn sowing, there was a trend toward higher plot-larval weight and lower single larval weight for treatments with more eggs inoculated. The soybean growth stage when eggs are inoculated and planting density significantly affect the growth of *C. bilineata tsingtauica* larvae in spring sowing. Inoculating eggs during the R3 stage and later or high planting density (≥19 plants/m^2^) will decrease larval yield in spring sowing. The larval number and plot-larval weight were the highest when the highly susceptible soybean variety NN89-29 was planted at a density of 15 plants/m^2^ with 25 eggs/m^2^ inoculated. In the V6 stage, the yield of larvae could reach 130.8 g/m^2^ in spring sowing. In autumn sowing, the susceptible variety NN89-29 at 15 plants/m^2^ with 25 eggs/m^2^ had relatively high-yielding production. Compared with spring sowing, autumn sowing with the same planting density, egg inoculate stage and egg-inoculate amount, the larval yield was low, and the plant biomass was small. So, the planting density should be appropriately increased to further improve the larval yield in autumn sowing. More affecting factors in *C. bilineata tsingtauica* artificial rearing need further study in the future. In conclusion, this study synthesized the appropriate levels of various factors influencing larval rearing, and these results proposed technical conditions for more efficiently rearing *C. bilineata tsingtauica* in spring and autumn sowing.

## Figures and Tables

**Table 1:** Analysis of variance of three factors of *C. bilineata tsingtauica* larval growth in spring sowing net house.

Source of Variation	*DF*	NL		SLW		PLW	
		*MS*	*F*	*MS*	*F*	*MS*	*F*
GS	1	570.4	7.9 *	58.7	83.1 **	231583.1	124.8 **
PD	2	1854	25.8 **	15.2	21.6 **	159702.5	86.1 **
NE	1	408.4	5.7 *	0.6	0.9	5651.3	3.1
GS × PD	2	129.5	1.8	2.9	4.2 *	30454.3	16.4 **
GS × NE	1	18.4	0.3	0	0	23.4	0.01
PD × NE	2	50	0.7	0.6	0.9	268.2	0.1
GS × PD × NE	2	24.5	0.3	0.5	0.7	1314.7	0.7
Error	12	72		0.7		1855	

Note: NL, number of larvae; SLW, single larval weight; PLW, plot-larval weight; GS, growth stage; PD, planting density; NE, number of eggs inoculated. * represents a 0.05 level of significance; ** represents significance at 0.01 probability level; *DF*, degrees of freedom; *MS*, mean square; *F*, *F* value.

**Table 2:** Analysis of variance of three factors of agronomic traits in spring sowing net house.

Source of Variation	*DF*	PH		MSN		IL	
		*MS*	*F*	*MS*	*F*	*MS*	*F*
GS	1	30988.9	109.9 **	18.0	14.8 **	37.2	62.5 **
PD	2	6318.6	22.4 **	11.6	9.6 **	23.2	39.0 **
NE	1	230.6	0.8	0.2	0.2	0.8	1.3
GS × PD	2	461.4	1.6	0.4	0.3	1.9	1.6
GS × NE	1	4.9	0	1.3	1.1	0.4	0.6
PD × NE	2	47.8	0.2	0.0	0.0	0.2	0.3
GS × PD × NE	2	10.5	0	0.5	0.4	0.1	0.1
Error	12	282.1		1.2		0.6	

Note: PH, plant height; MSN, main stem node number; IL, internode length; GS, growth stage; PD, planting density; NE, number of eggs inoculated. ** represents significance at 0.01 probability level; *DF*, degrees of freedom; *MS*, mean square; *F*, *F* value.

**Table 3:** Correlation analysis of growth indicators among soybean plants and *C. bilineata tsingtauica* in spring sowing net house.

Indicator	NL	SLW	PLW	PH	MSN	IL
NL		0.77	0.95 **	−0.76	0.70	−0.73
SLW	0.83 *		0.92 **	−0.82 *	0.85 *	−0.82 *
PLW	0.98 **	0.93 **		−0.79	0.79	−0.79
PH	−0.94 **	−0.93 **	−0.98 **		−0.96 **	0.99 **
MSN	0.78	0.82 *	0.86 *	−0.85 *		−0.98 **
IL	−0.94 **	−0.94* *	−0.99 **	0.99 **	−0.89 *	

Note: The lower left is data in the V6 stage, and the upper right is data in the R3 stage; NL, number of larvae; PLW, plot-larval weight; SLW, single larval weight; PH, plant height; MSN, main stem node number; IL, internode length; * represents significance at 0.05 probability level; ** represents significance at 0.01 probability level.

**Table 4:** Multiple comparisons of growth indicators of *C. bilineata tsingtauica* and soybean plants among different treatments in spring sowing.

Stage	PD (Plants/m^2^)	NE (Eggs/m^2^)	NL (Heads)	SLW (g)	PLW (g/m^2^)	PH (cm)	MSN	IL (cm)
V6	15	18.75	53.0 ± 0.71 abc	**9.4 ± 0.0 a**	124.8 ± 1.1 a	124.5 ± 1.1 d	23.4 ± 0.3 ab	5.3 ± 0.1 e
		25	**64.5 ± 0.35 a**	8.1 ± 0.1 ab	**130.8 ± 0.3 a**	127.1 ± 6.4 d	23.1 ± 0.9 ab	5.6 ± 0.5 e
	19	18.75	48.0 ± 5.66 abcd	8.2 ± 0.5 ab	97.1 ± 5.6 abc	146.1 ± 0.9 cd	21.1 ± 0.5 ab	6.9 ± 0.1 cde
		25	58.0 ± 2.12 ab	7.7 ± 0.2 abc	112.0 ± 1.7 ab	146.0 ± 12.7 cd	21.7 ± 0.5 ab	6.7 ± 0.4 de
	26	18.75	26.5 ± 1.06 cd	4.9 ± 0.6 cd	32.9 ± 5.0 de	188.5 ± 5.3 bc	20.4 ± 1.0 b	9.3 ± 0.7 abcd
		25	24.5 ± 5.30 cd	5.7 ± 0.7 bcd	36.5 ± 11.6 de	201.9 ± 14.9 abc	20.9 ± 0.6 ab	9.6 ± 0.4 abc
R3	15	18.75	42.5 ± 3.9 abcd	5.7 ± 0.2 bcd	60.3 ± 3.8 cd	206.4 ± 4.0 abc	**24.6 ± 0.1 a**	8.4 ± 0.1 bcd
		25	57.5 ± 0.4 ab	5.3 ± 0.1 bcd	75.8 ± 1.6 bc	213.3 ± 11.1 ab	24.5 ± 0.4 a	8.7 ± 0.3 bcd
	19	18.75	31.5 ± 4.6 bcd	3.8 ± 0.6 d	31.5 ± 9.4 de	220.5 ± 13.4 ab	24.0 ± 0.3 ab	9.2 ± 0.7 abcd
		25	38.0 ± 9.2 abcd	3.6 ± 0.7 d	31.1 ± 1.7 de	225.0 ± 7.2 ab	23.2 ± 0.3 ab	9.7 ± 0.2 abc
	26	18.75	19.0 ± 0.7 d	3.6 ± 0.3 d	17.1 ± 2.0 e	245.1 ± 0.4 ab	22.9 ± 0.2 ab	10.7 ± 0.1 ab
		25	27.5 ± 5.3 cd	3.3 ± 0.3 d	23.6 ± 6.4 de	**255.0 ± 4.5 a**	21.8 ± 0,7 ab	**11.7 ± 0.2 a**

Note: PD, planting density; NE, number of eggs inoculated; NL, number of larvae; SLW, single larval weight; PLW, plot-larval weight; PH, plant height; MSN, main stem node number; IL, internode length; underline represents the minimum value of each column; bold font represents the maximum value of each column. Different lowercase letters indicate significant differences of *p* < 0.05.

**Table 5:** Analysis of variance of *C. bilineata tsingtauica* larval growth indicators in autumn sowing net house.

Source of Variation	*DF*	NL		SLW		PLW		Proportion ^#^	
		*MS*	*F*	*MS*	*F*	*MS*	*F*	*MS*	*F*
NE	2	694.1	20.3 **	4.5	5.5 *	9498.5	6.5 *	62	11.2 **
UCPF	1	1.4	0.04	0.7	0.9	484.6	0.3	2837.8	0.3
NE × UCPF	2	125.7	3.67	1.4	1.7	2637.8	1.8	1181.6	4.7 *
Error	12	34.2		0.8		1466		3030.5	

Note: NL, number of larvae; SLW, single larval weight; PLW, plot-larval weight; NE, number of eggs inoculation; UCPF, uncovering or covering with plastic film; # represents the proportion of *C. bilineata tsingtauica* single larvae weigh more than or equal to 6 g (≥ 6 g) in each plot (net house); * represents significance at 0.05 probability level; ** represents significance at 0.01 probability level; *DF*, degrees of freedom; *MS*, mean square; *F*, *F* value.

**Table 6:** Analysis of variance of soybean agronomic traits in autumn sowing net house.

Source of Variation	*DF*	PH		MSN		IL	
		*MS*	*F*	*MS*	*F*	*MS*	*F*
NE	2	34.2	0.5	0.3	1.5	0.3	0.3
UCPF	1	5	0.1	0.4	2	0.6	0.5
NE × UCPF	2	163.5	2.3	0.3	1.3	0.8	0.8
Error	12	71.7		0.2		0.3	

Note: PH, plant height; MSN, main stem node number; IL, internode length; NE, number of eggs inoculated; UCPF, uncovering or covering with plastic film; *DF*, degrees of freedom; *MS*, mean square; *F*, *F* value.

## Data Availability

The raw data supporting the conclusions of this article will be made available by the corresponding author, without undue reservation.
